# Genetic Polymorphisms of *Stromal Interaction Molecule 1* Associated with the Erythrocyte Sedimentation Rate and C-Reactive Protein in HLA-B27 Positive Ankylosing Spondylitis Patients

**DOI:** 10.1371/journal.pone.0049698

**Published:** 2012-12-14

**Authors:** James Cheng-Chung Wei, Kuo-Sheng Hung, Yu-Wen Hsu, Ruey-Hong Wong, Chun-Huang Huang, Ming-Shiou Jan, Shyh-Jong Wu, Yung-Shun Juan, Wei-Chiao Chang

**Affiliations:** 1 Division of Allergy, Immunology and Rheumatology, Department of Medicine, Chung Shan Medical University Hospital, Taichung, Taiwan; 2 Institute of Medicine, Chung Shan Medical University, Taichung, Taiwan; 3 Department of Neurosurgery, Center of Excellence for Clinical Trial and Research, Graduate Institute of Injury Prevention and Control, Taipei Medical University, Wan Fang Medical Center, Taipei, Taiwan; 4 Department of Clinical Pharmacy, School of Pharmacy, Taipei Medical University, Taipei, Taiwan; 5 Department of Public Health, Chung Shan Medical University, Taichung, Taiwan; 6 Institute of Microbiology and Immunology, Chung Shan Medical University, Taichung, Taiwan; 7 Department of Medical Laboratory Science and Biotechnology, College of Health Sciences, Kaohsiung Medical University, Kaohsiung, Taiwan; 8 Department of Urology, Kaohsiung municipal Hsiao-Kang Hospital and College of Medicine, Kaohsiung Medical University, Kaohsiung, Taiwan; 9 Department of Pharmacy, Taipei Medical University-Wanfang Hospital, Taipei, Taiwan; Institut Jacques Monod, France

## Abstract

Ankylosing spondylitis (AS) is a chronic inflammation of the sacroiliac joints, spine and peripheral joints. The development of ankylosing spondylitis is still unclear. Genetics factors such as human leukocyte antigen *HLA-B27* and *ERAP1* have been widely reported to associate to AS susceptibility. In this study, we enrolled 361 AS patients and selected four tagging single nucleotides polymorphisms (tSNPs) at *STIM1* gene. The correlation between *STIM1* genetic polymorphisms and AS activity index (BASDAI, BASFI, BAS-G) as well as laboratory parameters of inflammation (erythrocyte sedimentation rate (ESR) and C-reactive protein (CRP)) were tested. Our results indicated that HLA-B27 positive AS patients who are carrying the minor allele homozygous G/G genotype of SNP rs3750996 significantly associated with a higher level of ESR in serum. Furthermore, rs3750996/rs3750994 pairwise allele analysis indicated that G-C haplotypes also significantly correlated with higher level of ESR as well as CRP. These findings provide a better understanding of *STIM1* genetic contribution to the pathogenesis of AS.

## Introduction

Ankylosing spondylitis (AS) is a chronic inflammatory disorder of the lumbar spine and sacroiliac that can also affect the peripheral joints [1]. Males are affected more frequently than females [2]. AS strongly associates with the human leukocyte antigen (*HLA*)*-B27* gene [3], but *HLA-B27* accounts for only 16% of the genetic variability in AS [4]. HLA-B60, B61 and IL-1, and IL-23R genes also have been proven to be important in the pathogenesis of AS [5]–[7]. In 2010, Lee et al. [8] identified that *CTLA-4* +49A>G genotype associated with circulatory CRP level. These results indicated that the level of inflammation in AS subjects may be pre-determined by *CTLA-4* genotypes.

Our previous studies indicated a significant association between genetic polymorphisms of store-operated calcium channel, *ORAI1*, and the risk of inflammatory diseases such as HLA-B27 positive AS and calcium nephrolithiasis [9], [10]. In non-excitable cells such as T cell and mast cell, calcium influx is mainly via store-operated calcium channels (SOC) [11]. SOC is involved in a variety of physiological processes such as gene transcription, enzyme metabolism and inflammatory reaction. The regulation mechanism of store-operated calcium entry was unclear until 2005, Roos et al, firstly identified a molecule called Stromal interaction molecule 1 (STIM1) [12]. STIM1 is a calcium sensor that localized in the endoplasmic reticulum. Upon activation of IP_3_ receptor, calcium concentration in the store falls, which triggers the aggregation of STIM1, that resulted in the activation of store-operated calcium channel. Aberrant expression of STIM1-mediated calcium signaling has been implicated in the development of human cancers [13], [14]. Knockdown *STIM1* by siRNA which impairs Ca^2+^ influx, prevents the translocation of transcription factors and subsequent inflammatory *COX-2* gene activation [15], [16].

In this study, we investigated the association between *STIM1* genetic polymorphisms, AS activity index (BASDAI, BASFI, BAS-G) and inflammatory biochemical examines (ESR and CRP). Our results indicated that rs3750996 in the *STIM1* gene significantly associated with a higher level of ESR. Furthermore, G-C haplotypes (rs3750996/rs3750994) significantly correlated with higher level of ESR and CRP. These findings provide a better understanding of *STIM1* genetic contribution to the pathogenesis of AS.

## Materials and Methods

### Patients studied

Patients were solicited sequentially at Chung Shan Medical University Hospital in Taichung, Taiwan. AS patients who met selection criteria were asked to participate in the study. Informed consent was obtained before any data was collected from the respondents. Three selection criteria were used to recruit AS patients: (a) patients aged 16–65 years; (b) AS diagnosis by the modified New York criteria [17]; and (c) cognitive performance not influenced by other diseases such as dementia. Sacroilitis was confirmed by a qualified radiologist and AS diagnosis by a qualified rheumatologist. The detailed clinical history included age on initial symptom, family history of AS, and extraspinal manifestations. Age of AS symptom onset was defined as the time when the first symptom (axial symptom, peripheral arthritis, uveitis or enthesitis) had developed. Peripheral arthritis was defined as the presence of at least one swollen joint. Inflammatory bowel disease (IBD) (distinct from irritable bowel syndrome) was defined as the presence of the inflammatory condition of the colon and small intestine, including ulcerative colitis and Crohn's disease. Uveitis was defined as the presence of inflammation of the middle layer of the eye and involved patterns as unilateral, bilateral, or alternative. These symptoms were ascertained by the rheumatologist, ophthalmologist and gastroenterologist, and were recorded in medical record reviews. 100% of AS patients in this study have sacroiliitis. The design of the work and final report conformed to the Declaration of Helsinki and study was approved by the Institute Review Board of Chung Shan Medical University Hospital. All the subjects gave the written consent form.

### Bath Ankylosing Spondylitis Indices

The Bath Ankylosing Spondylitis Disease Activity Index (BASDAI), Bath Ankylosing Spondylitis Functional Index (BASFI) and Bath Ankylosing Spondylitis Global (BAS-G) were applied to evaluate the disease activity, physical function and global wellbeing, respectively. The modified Chinese versions of BASDAI, BASFI, and BAS-G have good intra-class correlation and Cronbach's alpha [18].

### Laboratory analyses

Peripheral blood was collected, and was centrifuged to separate the serum and the cells. Erythrocyte sedimentation rate (ESR), and C-reactive protein (CRP) were measured. HLA-B27 carriage was assessed by flow cytometry [19].

### DNA extraction

Blood cells were subjected to DNA extraction by treating them first with 0.5% SDS lysis buffer and then protease K (1 mg/ml) for digestion of nuclear protein for 4 h at 60°C. Total DNA was harvested by using the Gentra extraction kit followed by 70% alcohol precipitation.

### Genotyping

**Figure 1:**
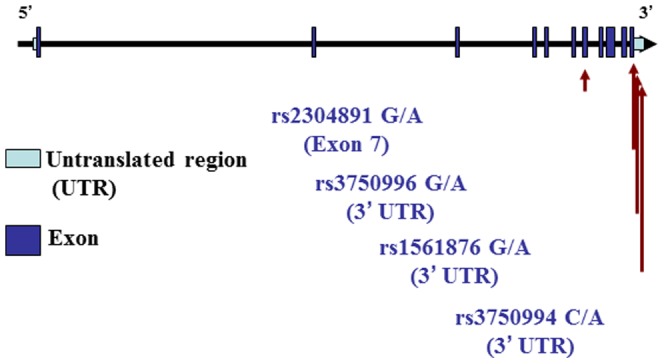
Graphical overview of the genotyped human *STIM1* gene polymorphisms in relation to its exon/intron structure.

Four tagging SNPs of *STIM1* (rs2304891, rs3750996, rs1561876, rs3750994) with a minimum allele frequency of greater than 10% in the Han Chinese in Beijing population were selected from the HapMap database (http://hapmap.ncbi.nlm.nih.gov/). A graphical overview of genotyped polymorphisms is shown in **Figure 1**. One polymorphism (rs2304891) of *STIM1* located in the exon, other three polymorphisms are in the 3′ untranslated region (UTR).Genotyping was carried out using the TaqMan Allelic Discrimination Assay (Applied Biosystems, Foster city, CA) as our previous report [10]. The polymerase chain reaction (PCR) was performed by using a 96-well microplate with the ABI9700 Thermal Cycler. After PCR, fluorescence was detected and analyzed using the System SDS software version 1.2.3.

### Cell culture

THP-1 cells were bought from ATCC. Cells were cultured (37°C, 5% CO_2_) in RPMI-1640 medium (GIBCO), supplemented with 10% fetal bovine serum and 10% penicillin-streptomycin.

### Reverse transcriptase PCR determination of TNF-α expression in THP-1 cells

Total RNA was extracted from THP-1 cells by RNeasy Mini Kit (Qiagen). A reverse transcriptase reaction was performed on 1 µg of extracted total RNA using reverse transcriptase reaction Kit (Applied Biosystems) according to the manufacturer's instructions. Following cDNA synthesis, Real-time PCR was performed in triplicate using a SYBR Green Master Mix. The specific primer of TNF-α were forward primer: 5′-GACAAGCCTGTAGCCCATGTTGTA-3′ and reverse primer: 5′-CAGCCTTGGCCCTTGAAGA-3′. Each well contained the following reaction mix: 2 µl cDNA, 5 µl 10× Sensimix dT (Quantace, Watford, UK), 2.8 µl RNase-free water (QIAGEN), 0.1 µl forward primer, and 0.1 µl reverse primer. Universal cycling conditions were used (one cycle at 95°C for 15 min and 40 cycles at 90°C for 15 s and 60°C for 60 s). Relative gene expression was calculated using the comparative CT method. All values were normalized to the housekeeping gene.

### Transfection of siRNA

Cells were seeded in 6-well plates one day before transfection. The *STIM1* siRNA was purchased from santa cruz biotechnology, Inc. *STIM1* siRNA was transfected into cells by using lipofectamine 2000 (Invitrogen). Following transfection, the cells were cultured for 24 h and then prepare for Thapsigargin (2 uM) stimulation.

### Measurement of IL-6 and TNF-α

IL-6 (Invitrogen Corp. CA, USA) and TNF- α (Invitrogen Corp. CA, USA) assays were performed by using enzyme linked immuno sorbent assay method (ELISA) (Tecan Minilyser, Tecan Group Ltd. Mannedorf, Switzerland). IL-6 and TNF- α assay measurements were carried out at 450 nm optical density (OD). Samples were analyzed in triplicate, and mean concentrations were calculated for each sample.

### Statistical analysis

JMP 8.0 for Windows was used for analysis. Analysis of variance (ANOVA) was used to compare the mean of continuous variables (BASDAI, BASFI, BAS-G, ESR and CRP) among different genotypes in AS patients. Multiple regression analysis was used to adjust for age, sex and disease duration. A *P* value less than 0.05 is considered significant. Linkage disequilibrium (LD) was assessed for any pair of SNPs and haplotype blocks were defined using the default setting of the Haploview software 4.2 (Broad Institute, Cambridge, Massachusetts) and PHASE version 2.1.

## Results

### Basic and Clinical Characteristics of the Subjects

A total 361 AS patients were recruited in this study. **Table 1** showed the characteristics of the subjects. 67.9% of cases were male. The mean age (years) and standard deviation (S.D.) were 33.5±12.8. In AS subjects, 87.3% (315/361) were HLA-B27 positive and their mean BASDAI, mean BASFI, and mean BAS-G scores were 4.1±2.3, 1.9±2.2, and 4.3±2.8, respectively.

**Table 1:** Basal characteristics and clinical features of patients with ankylosing spondylitis (AS).

Characteristics	Patients with AS
Number of subjects	361
Gender:male, No (%)	245 (67.9%)
Age (years)^a^	33.5±12.8
Range	6–69
HLA-B27(+)	315 (87.3%)
BASDAI (0-10)	4.1±2.3
BASFI (0-10)	1.9±2.2
BAS-G (0-10)	4.3±2.8

a Mean ± SD. SD:standard deviation.

### Association of *STIM1* genetic polymorphisms with the rate of ESR in HLA-B27 positive AS patients

We analyzed the relationship between disease activity index (BASDAI, BASFI and BAS-G) and the four polymorphisms of *STIM1* among HLA-B27 positive AS patients. A borderline significant association between *STIM1* polymorphism rs1561876 and BASFI (*P*-value = 0.04) or BAS-G (*P*-value = 0.06) was found. However, we failed to improve the significance even after adjustment for the effects of ages and sex (**Table 2**). We further analyzed the association between inflammatory biochemical examination (ESR and CRP) and *STIM1* gene polymorphisms. As shown in **Table 3**, rs3750996 homozygous G/G genotype significantly correlated with increased level of ESR compared with the A/G and A/A genotypes in HLA-B27 positive AS patients (*P*-value = 0.01). In addition, the risk G allele of rs3750996 in HLA-B27 positive AS patients was seen in a higher CRP level (*P*-value = 0.06).

**Table 2:** Difference in the scores of BASDAI, BASFI, and BAS-G among HLA-B27 positive AS patients stratified by different *STIM1* genotype.

SNP	Genotype	Number (%)	BASDAI	BASFI	BAS-G
rs2304891	GG	58 (19.2)	4.2±2.3^a^	1.6±2.1	3.7±2.7
	AG	134 (44.4)	4.3±2.4	2.1±2.4	4.5±2.9
	AA	110 (36.4)	3.9±2.2	1.9±2.1	4.2±2.9
Unadjusted *P*-value			0.58	0.49	0.31
Adjusted *P*-value			0.58†	0.52§	0.31†
rs3750996	GG	13 (4.3)	3.4±2.3	1.7±1.7	3.3±2.4
	AG	104 (34.6)	4.2±2.2	1.9±2.2	4.5±2.8
	AA	184 (61.1)	4.0±2.3	1.9±2.2	4.0±2.9
Unadjusted *P*-value			0.42	0.98	0.22
Adjusted *P*-value			0.43†	0.97§	0.21†
rs1561876	GG	23 (7.8)	3.6±1.9	1.7±1.8	3.7±2.9
	AG	118 (40.0)	4.3±2.3	2.4±2.5	4.7±3.0
	AA	154 (52.2)	3.9±2.4	1.6±2.0	3.9±2.8
Unadjusted *P*-value			0.38	0.04*	0.06
Adjusted *P*-value			0.38†	0.03§ *	0.06†
rs3750994	CC	18 (5.9)	3.9±2.6	1.9±2.2	4.5±3.1
	AC	116 (38.2)	4.1±2.3	2.0±2.2	4.2±3.0
	AA	170 (55.9)	4.1±2.3	1.9±2.3	4.3±2.7
Unadjusted *P*-value			0.93	0.92	0.90
Adjusted *P*-value			0.93†	0.93§	0.90†

a Data represent means ± S.D..

† Adjusted the effects of age and sex.

§ Adjusted the effects of age, sex and disease duration.

* Significant (*P*<0.05) values are in bold.

**Table 3:** Difference in the value of ESR and CRP among HLA-B27 positive AS patients stratified by different *STIM1* genotype.

SNP	Genotype	Number (%)	ESR	CRP
rs2304891	GG	58 (19.2)	20.4±14.9^a^	0.8±1.3
	AG	134 (44.4)	23.1±19.4	1.3±1.9
	AA	110 (36.4)	25.2±20.2	1.5±2.3
Unadjusted *P*-value			0.38	0.14
Adjusted *P*-value†			0.38	0.12
rs3750996	GG	13 (4.3)	45.3±26.1	3.0±3.0
	AG	104 (34.6)	22.3±19.7	1.2±2.0
	AA	184 (61.1)	22.4±17.3	1.2±1.9
Unadjusted *P*-value			**0.01** *	0.06
Adjusted *P*-value†			**0.01** *	0.05
rs1561876	GG	23 (7.8)	25.8±17.7	1.1±1.9
	AG	118 (40.0)	21.8±18.7	1.4±2.2
	AA	154 (52.2)	24.4±18.9	1.2±1.9
Unadjusted *P*-value			0.53	0.80
Adjusted *P*-value†			0.53	0.79
rs3750994	CC	18 (5.9)	20.4±13.3	0.6±0.9
	AC	116 (38.2)	21.8±18.4	1.2±2.0
	AA	170 (55.9)	25.0±19.6	1.4±2.0
Unadjusted *P*-value			0.38	0.49
Adjusted *P*-value†			0.38	0.46

a Data represent means ± S.D..

† Adjusted the effects of age and sex.

* Significant (*P*<0.05) values are in bold.

### *STIM1* Haplotypes associated with ESR and CRP levels in HLA-B27 positive AS patients

**Figure 2:**
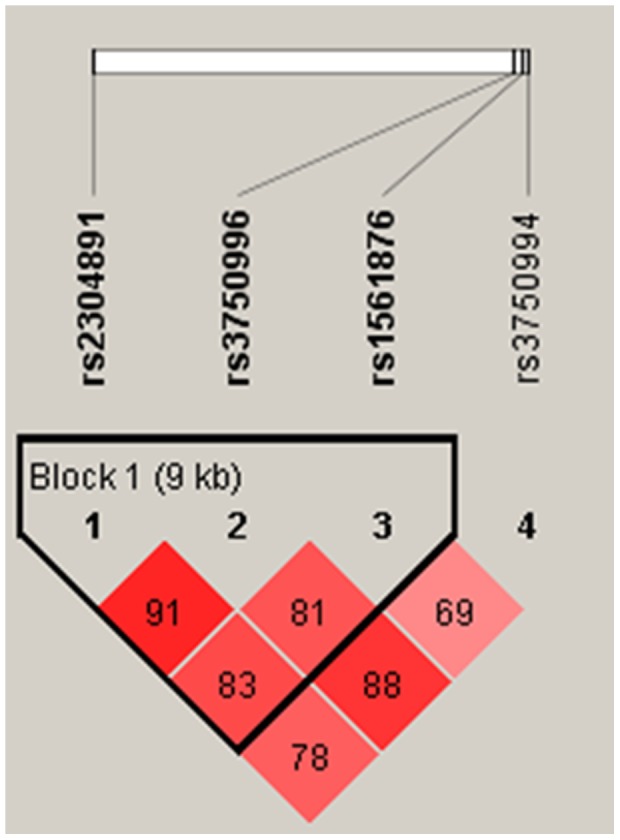
*STIM1* gene LD and haplotype block structure in HLA-B27(+) AS. The number on the cells is the LOD score of D′.

We further calculated pairwise linkage disequilibrium (LD) (**Figure 2**) and analyzed two common haplotypes by using the Haploview 4.2 program and PHASE version 2.1. As shown in the **Table 4**, haplotypes of rs3750996/rs3750994 is significantly associated with ESR in the HLA-B27 positive AS patients (*P* = 0.03). In addition, rs3750996/rs3750994 haplotype G/C patients had higher CRP level (*P* = 0.001) (**Table 5**). After adjustment for the effects of age and gender, the significant association still exists (**Table 4** **and** **Table 5**).

**Table 4:** Difference in the value of ESR among HLA-B27 positive AS patients stratified by different *STIM1* haplotypes.

rs2304891/rs3750996	ESR	rs3750996/rs1561876	ESR	rs3750996/rs3750994	ESR
G/G	41.0±4.2^a^	G/G	14.5±4.9	G/C	72.0
A/G	25.2±22.0	A/G	22.8±18.5	G/A	25.0±21.5
G/A	21.6±17.4	G/A	25.8±22.2	A/C	21.1±17.0
A/A	23.7±18.5	A/A	22.3±16.9	A/A	23.2±18.2
Unadjusted *P*-value	0.23	Unadjusted *P*-value	0.43	Unadjusted *P*-value	**0.03** *
Adjusted *P*-value†	0.23	Adjusted *P*-value	0.42	Adjusted *P*-value†	**0.03** *

a Data represent mean ± S.D..

† Adjusted the effects of age and sex.

* Significant (*P*<0.05) values are in bold.

**Table 5:** Difference in the value of CRP among HLA-B27 positive AS patients stratified by different *STIM1* haplotypes.

rs2304891/rs3750996	CRP	rs3750996/rs1561876	CRP	rs3750996/rs3750994	CRP
G/G	1.0±0.6^a^	G/G	0.2±0.3	G/C	8.6
A/G	1.5±2.2	A/G	1.3±2.1	G/A	1.4±2.1
G/A	1.1±1.7	G/A	1.4±2.2	A/C	1.1±1.7
A/A	1.4±2.1	A/A	1.2±1.8	A/A	1.3±2.0
Unadjusted *P*-value	0.36	Unadjusted *P*-value	0.64	Unadjusted *P*-value	**0.001** *
Adjusted *P*-value†	0.32	Adjusted *P*-value	0.65	Adjusted *P*-value†	**0.001** *

a Data represent mean ± S.D..

† Adjusted the effects of age and sex.

* Significant (*P*<0.05) values are in bold.

### Association of *STIM1* genotypes and cytokines (TNF-α and IL-6) levels

**Figure 3:**
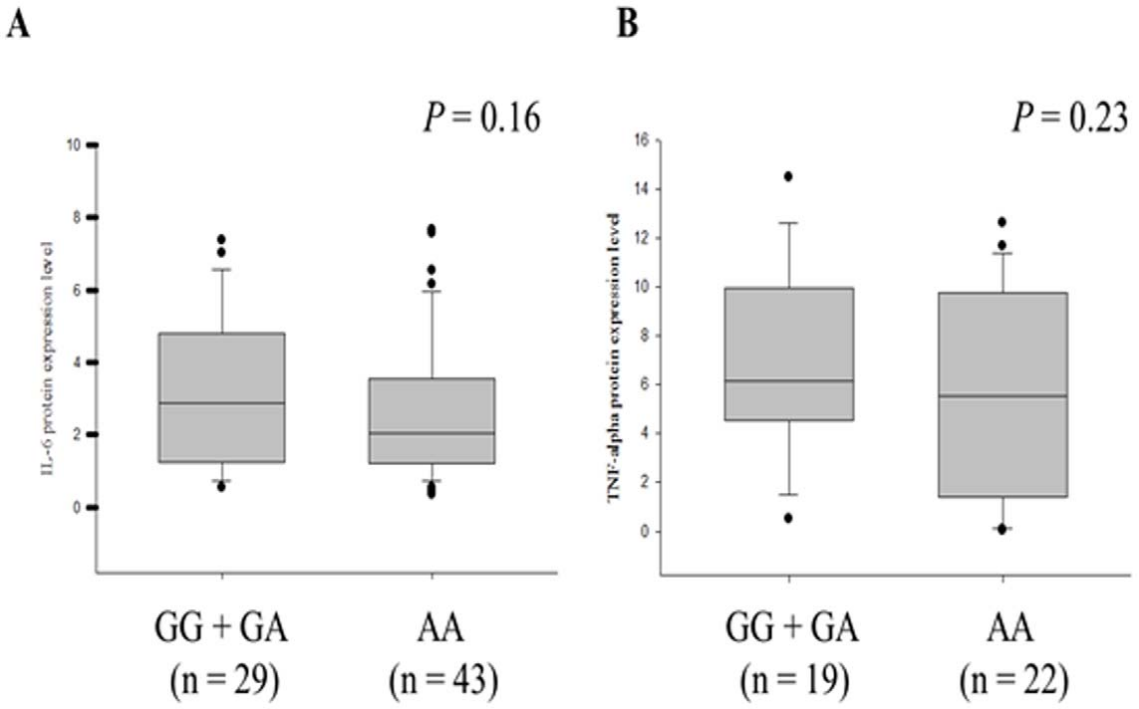
Comparison of serum (A) IL-6 and (B) TNF-α levels among different genotypes of *STIM1* (rs3750996) in AS patients.

Gratacós et al., indicated that cytokines (TNF-α and IL-6) are increased in AS patients [20]. Expression level of IL-6 strongly correlated with clinical parameters of inflammation such as ESR and CRP. Thus, we further test the functional correlation between *STIM1* genotypes and cytokine (IL-6 and TNF-α). As shown in the **Fig. 3**, the AS patients with G/G or G/A genotypes of the *STIM1* showed a profound increase of serum IL-6 and TNF-α. AS patients with AA homozygote, however, has a lower level of IL-6 and TNF-α.

## Discussion

Acute phase reactants, including ESR and CRP, are generally used to evaluate AS patients and are also recommended core set endpoint for disease controlling antirheumatic therapy (DC-ART) [21]. Ruof et al. [22] observed a strong correlation between ESR and CRP. Yildirim et al., provided evidence for a close association between CRP and BASDAI [23]. In addition, in large prospective cohort study for in AS patients, ESR and CRP are served as powerful tools not only for monitoring the efficacy of anti-TNF therapy, but also for the selection of AS patients with a high likelihood of responding to anti-TNF treatment [24]. Therefore, the level of ESR and CRP has been widely used in the clinical diagnosis as well as treatment in AS patients.

The genetic polymorphism of Vitamin D receptor (*FokI*) was associated with the levels of ESR and CRP in AS patients [25]. In a Taiwanese population, Lee et al., reported that genotypes of *cytotoxic T lymphocyte-associated antigen-4 (CTLA-4)* associated with expression level of CRP in AS patients [8]. In this study, our results revealed a strong correlation between *STIM1* genotypes/haplotypes and the level of inflammatory factors (ESR and CRP). Since store-operated calcium entry is important in T cell-mediated autoimmunity [26], our results implied that polymorphisms of *STIM1* may influence store-operated calcium signals which in turn involve the regulation of cytokine release and ESR/CRP expression. Indeed, the AS patients with G/G and G/A genotypes of the *STIM1* polymorphism showed a higher level of serum IL-6 and TNF-α. Although the *P* value (0.16; 0.23) was still not significant, we attribute this result to the reduction of the sample size (only 72 cases with IL-6 data and 41 cases with TNF-α data).

In non-excitable cells such as T cells and mast cells, one major route for Ca^2+^ entry is through store-operated Ca^2+^ channels. Store-operated calcium entry has been reported to regulate paracrine (LTC_4_) signals in mast cells and autoimmunity in T cells [27], [28]. In T cells [26], [29], STIM1 is a key initiator that involves in the activation of store-operated Ca^2+^ entry. Picard et al., reported a homozygous nonsense mutation in the *STIM1* gene the caused the deficiency of Ca^2+^ entry which leads to immune dysfunction [30]. In B cells, STIM1-mediated calcium signals drive translocation of Ca^2+^-dependent transcription factor NF-AT to the nucleus where it triggers *interleukin (IL)-10* gene [31]. Thus, STIM1 is an important regulator for cytokine production. Using cell-based experiments, our studies also indicated that knockdown *STIM1* resulted in the reduction of thapsigangin-mediated *TNF-α* expression (supplementary Fig 1). Therefore, polymorphisms of *STIM1* are very likely to involve in the regulation of immune system, which in turn control the ESR/CRP levels. Even so, the mechanism of stim1-mediated ESR/CRP pathways remains to be elucidated.

rs3750996 is located in the 3′UTR of *STIM1* gene. The mechanism by which miRNAs regulate *STIM1* gene expression is still unclear. By bioinformatics approaches from miRBase (http://www.mirbase.org), the allele variations on rs3750996 position may influence the binding affinity of miR223. The molecular mechanism of how miR223 regulates *STIM1* expression needs to be further investigated.

We also analyzed the relationship between genetic polymorphism rs3750996 and ESR in the HLA-B27 negative AS patients (46 HLA-B27 negative AS patients), however, no statistically significant association between genotypes and phenotypes were found (data not shown). We acknowledged that the tSNPs (exon and UTR) selected in this study may be not adequate to investigate the entire genetic polymorphisms of *STIM1*. Application of direct sequencing in a larger sample size may be helpful to identify novel polymorphisms of *STIM1*. In conclusion, our research indicated a significant association between genetic polymorphisms of *STIM1* and ESR/CRP in the HLA-B27 positive AS patients. Haplotypes of rs3750996/rs3750994 also further confirm the association.

## Supporting Information

Figure S1 **Knockdown** ***STIM1*** **gene reduced Thapsigargin-mediated** ***TNF-α*** **gene expression in THP-1 cells.**
(TIF)
