# Supplementary figures and images for: Genetic Polymorphisms of Stromal Interaction Molecule 1 Associated with the Erythrocyte Sedimentation Rate and C-Reactive Protein in HLA-B27 Positive Ankylosing Spondylitis Patients
Source: PLoS One. 2012 Dec 14;7(12):e49698. doi: 10.1371/journal.pone.0049698 (PMC3522685; doi:10.1371/journal.pone.0049698)

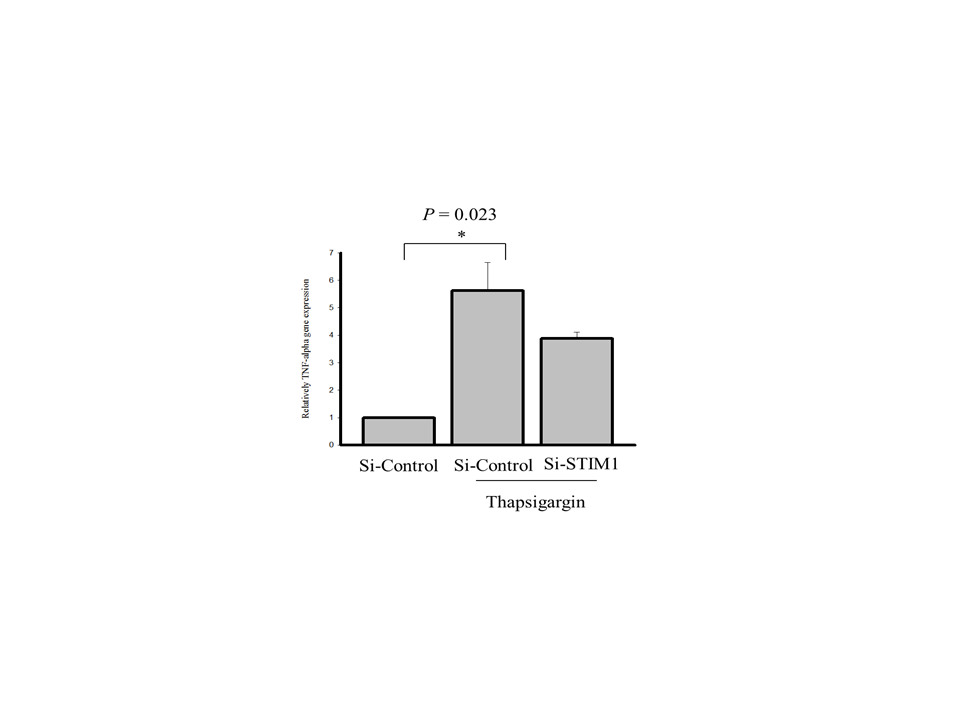

Supplement: Figure S1 — Knockdown STIM1 gene reduced Thapsigargin-mediated TNF-α gene expression in THP-1 cells. (TIF) [file pone.0049698.s001.tif]
